# Data on leaf structural, physiological and nutritional characteristics of species co-occurring in restinga and semidecidual seasonal forest ecosystems

**DOI:** 10.1016/j.dib.2020.105484

**Published:** 2020-04-08

**Authors:** Saulo Pireda, Dhiego da Silva Oliveira, Neilor Lacorte Borges, Gabriel do Amaral Ferreira, Laura Mathias Barroso, Priscila Simioni, Ângela Pierre Vitória, Maura Da Cunha

**Affiliations:** a Universidade Estadual do Norte Fluminense Darcy Ribeiro, Laboratório de Biologia Celular e Tecidual, Centro de Biociências e Biotecnologia. Brazil; b Universidade Estadual do Norte Fluminense Darcy Ribeiro, Laboratório de Entomologia e Fitopatologia, Campos dos Goytacazes, RJ, Brazil; c Universidade do Estado do Rio de Janeiro, Laboratório de Anatomia Vegetal. Brazil; d Universidade Estadual do Norte Fluminense Darcy Ribeiro, Laboratório de Ciências Ambientais, Centro de Biociências e Biotecnologia. Brazil

**Keywords:** Leaf anatomy, Gas exchange, Chlorophyll a fluorescence, Nutritional analysis, Atlantic forest

## Abstract

This paper presents additional data on the leaf structural, physiological and nutritional characteristics of three species (*Maytenus obtusifolia, Manilkara subsericea* e *Inga laurina*), co-occurring in restinga and semideciduous seasonal forest (forest). The data of the leaf structural, physiological and nutritional characteristics were obtained from the three species to identify possible adaptive strategies that could explain the co-occurrence of these species in the restinga and forest. In addition, this data can help identify key functional traits in the plant community of restinga and forests that can be employed in the reestablishment of ecological and edaphic processes in these ecosystems. This work presents data complementary to the published article “Acclimatization capacity of leaf traits of species co-occurring in restinga and seasonal semideciduous forest ecosystems” [1]

Specifications table

**Table:** 

Subject	Agricultural and Biological Sciences/Plant Science
Specific subject area	Anatomy, ecophysiology and nutrition leaf of plant species from the Atlantic Forest
Type of data	Additional data on the anatomical, physiological and nutritional characteristics of the leaves of native species of the Atlantic Forest
How data were acquired	Light microscopy (Axioplan, ZEISS, Germany); infrared carbon dioxide analyzer (LCpro-SD, ADC BioScientific Ltd., UK); portable fluorimeter (OS5p Opti-Sciences, UK).
Data format	Raw, graph, table and figure
Parameters for data collection	Climatic and seasonal conditions.
Description of data collection	Data were collected on sunny days and during the summer (January and February)
Data source location	Laboratory of Cell Biology and Tissue, Universidade Estadual do Norte Fluminense Darcy Ribeiro - Brazil.
Data accessibility	The raw data files are provided in the Mendeley Data, http://dx.doi.org/10.17632/tm45d63jrc.1. All other data is with this article
Related research article	Pireda et al. (2019). Acclimatization capacity of leaf traits of species co-occurring in restinga and seasonal semideciduous forest ecosystems. Environ. Exp. Bot. 164: 190–202. https://doi.org/10.1016/j.envexpbot.2019.05.012

## Value of the data

• There is no description for the leaf anatomical characteristics of *M. obtusifolia, M. subsericea* and *I. laurina* in the literature. In this sense, this data can provide new information regarding the taxonomy, ontogenetic and ecological researches of these species.
• Ecophysiological data on native species of the Atlantic Forest are scarce. Thus, these data may attract the attention of other researchers who work with ecophysiology of native species.
• The data on the nutritional composition of leaves and physical-chemical characteristics of the soil may serve as a reference for other works developed within restingas and semideciduous seasonal forests.
• Anatomical and physiological differences presented in this paper, show that the species need to adjust their phenotypic characteristics to co-occur in restinga and semidecidual seasonal forest. This information is important to encourage further investigations on the phenotypic plasticity of native species of the Atlantic Forest.
• Preliminary and detailed identification of key functional traits in the plant community of restinga and semideciduous seasonal forests can increase the probability of success in ecological restoration actions and in the reestablishment of ecological and edaphic processes in these ecosystems.

## 1 Data

**Fig. 1:**
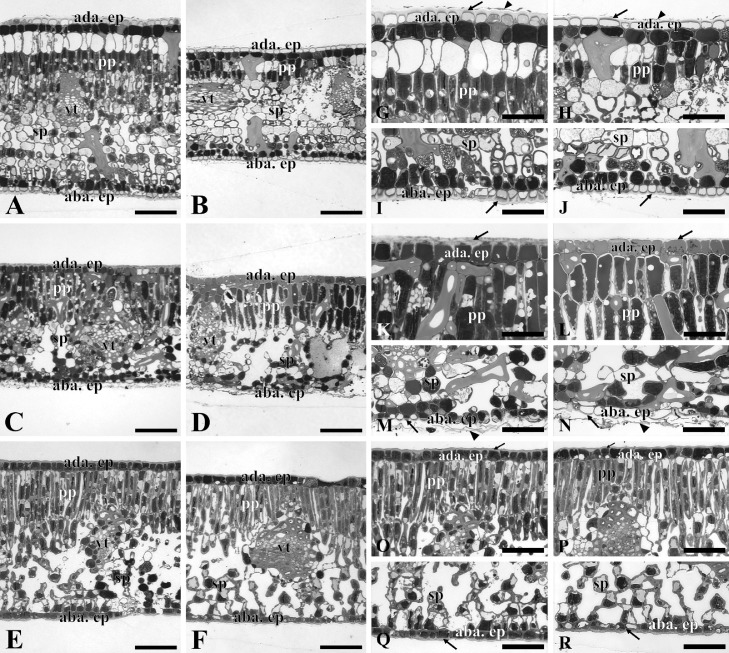
Leaf anatomical characterization of *Maytenus obtusifolia, Manilkara subsericea* and *Inga laurina* observed in light microscopy. Leaf blade of *M. obtusifolia* in restinga (A) and forest (B); Leaf blade of *M. subsericea* in restinga (C) and forest (D); Leaf blade of *I. laurina* in restinga (E) and forest (F); Highlight of the adaxial epidermis of *M. obtusifolia* in restinga (G) and forest (H); Highlight of the abaxial epidermis of *M. obtusifolia* in restinga (I) and forest (J); Highlight of the adaxial epidermis of *M. subsericea* in restinga (K) and forest (L); Highlight of the abaxial epidermis of *M. subsericea* in restinga (M) and forest (N); Highlight of the adaxial epidermis of *I. laurina* in restinga (O) and forest (P); Highlight of the abaxial epidermis of *I. laurina* in restinga (Q) and forest (R). ada ep – adaxial epidermis; aba ep – abaxial epidermis; pp – palisade parenchyma; sp – spongy parenchyma; vt – vascular tissue; arrows indicate the cuticle thickness; arrowhead indicate the presence of epicuticular wax. Bars: A – F: 100 µm; G – R: 50 µm.

**Fig. 2:**
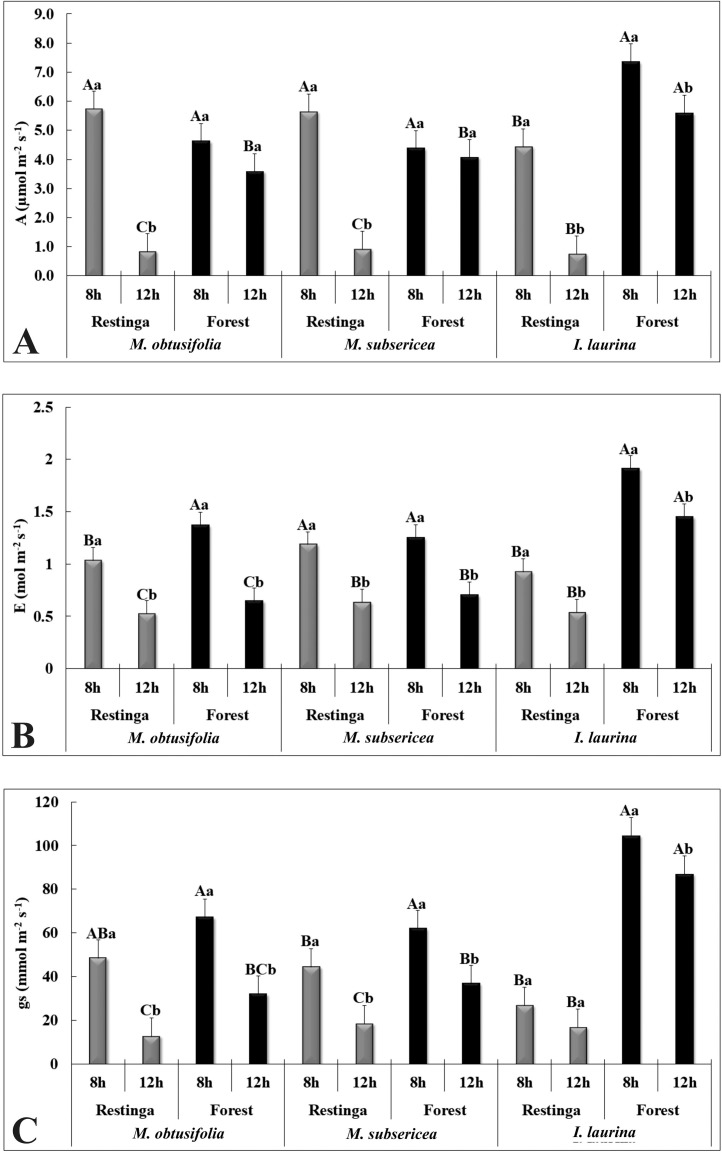
Mean values (± standard deviation) of the gas exchange performed during the day (8h and 12h) for the species *M. obtusifolia, M. subsericea* and *I. laurina* in restinga and forest. A – Liquid photosynthetic rate (*A* µm*ol m^−2^ s^−1^*). B – Transpiration rate (*E* mol *m^−2^ s^−1^*). C – Stomatal conductance (*gs* mmol *m^−2^ s^−1^*). Different letters indicate significant differences between ecosystems. Lowercase letters indicate significant differences between times while uppercase letters indicate differences between sites (Tukey *p*≤0.05).

**Fig. 3:**
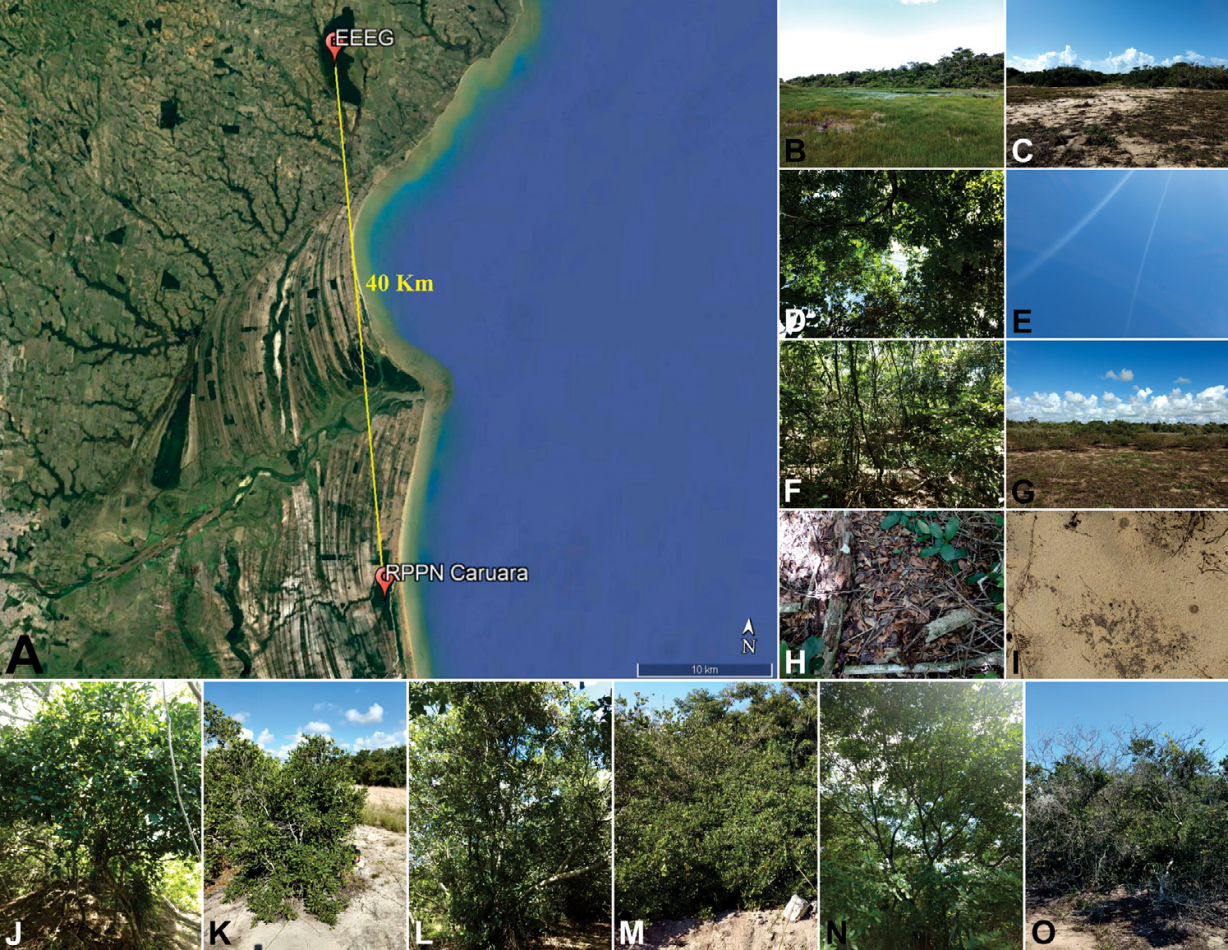
Characterization of the sampling location and habit of the three species. A – Overview of the sites highlighting the straight-line distance between RPPN Fazenda Caruara (restinga) and EEEG (forest) (Google Earth). B – Overview of the forest site, highlighting the presence of a lake in the surroundings. C – Overview of the restinga site. D – Detail of the canopy in the forest site. E – Absence of a canopy in the restinga site. F – Detail of understory in the forest site, highlighting the great diversity of species. G – Absence of understory in the restinga site. H – Detail of the soil in the forest site, highlighting the presence of litter. I – Detail of soil in the restinga site, highlighting its sandy composition. Detail of *M. obtusifolia* species in the forest (J) and in the restinga (K); *M. subsericea* in the forest (L) and in the restinga (M); and *I. laurina* in the forest (N) and in the restinga (O). Figures B – O: personal archive.

The dataset in this paper demonstrates changes in the anatomical, physiological and nutritional characteristics of the leaves of three species (*Maytenus obtusifolia, Manilkara subsericea* e *Inga laurina*) that co-occur in two ecosystems of the Atlantic Forest: restinga and semideciduous seasonal forest (SSF). In addition, this work presents the nutritional and physical characteristics of the soil of restinga and SSF. The Fig. 1 shows images of the leaf blade obtained through light microscopy. The Fig. 2 show the gas exchange data carried out during the day (8 am and 12 pm) in restinga and SFF, from which were evaluated the liquid photosynthetic rate (*A* µmol m*^−2^* s*^−1^*), transpiration rate (*E mol* m*^−2^* s*^−1^*), stomatal conductance (gs mmol m*^−2^* s*^−1^*). The Table 1 show the data from the chlorophyll *a* fluorescence performed during the day (8h and 12h) in restinga and SFF. For this, the quantum yield of PSII (*YII*) and maximum quantum yield of PSII (*Fv/Fm*) were evaluated. The Table 2 show the data of the nutritional content in leaves of the three species in restinga and SSF. The Table 3 show the data of the nutritional and physical-chemical characteristics of the soil in restinga and SSF. The Fig. 3 show images from where the samples were collected and the habit of the three species.

**Table 1:** Mean values (± standard deviation) of the chlorophyll *a* fluorescence performed during the day (8h and 12h) for the species *M. obtusifolia, M. subsericea* and *I. laurina* in restinga and forest. The following variables were obtained: quantum yield of PSII (*YII*) and maximum quantum yield of PSII (*Fv/Fm*). Different letters indicate significant differences to ecosystems. Lowercase letters indicate differences between hours while uppercase letters indicate significant difference between sites. (Tukey p≤0.05).

Species	Ecosystem	Hour	Traits	
			*Y(II)*	*Fv/Fm*
*M. obtusifolia*	Restinga	8:00h	0.41±0.13 **Ba**	0.77±0.13 **Aa**
		12:00h	0.34±0.10 **Bb**	0.77±0.10 **Aa**
	Forest	8:00h	0.75±0.01 **Aa**	0.77±0.01 **Aa**
		12:00h	0.68±0.03 **Ab**	0.78±0.03 **Aa**
*M. subsericea*	Restinga	8:00h	0.40±0.11 **Ba**	0.77±0.11 **Aa**
		12:00h	0.23±0.08 **Bb**	0.76±0.08 **Ba**
	Forest	8:00h	0.69±0.02 **Aa**	0.78±0.02 **Aa**
		12:00h	0.67±0.03 **Aa**	0.79±0.03 **Aa**
*I. laurina*	Restinga	8:00h	0.43±0.17 **Ba**	0.74±0.17 **Ba**
		12:00h	0.26±0.09 **Bb**	0.72±0.09 **Ba**
	Forest	8:00h	0.55±0.10 **Aa**	0.79±0.10 **Aa**
		12:00h	0.58±0.08 **Aa**	0.79±0.08 **Aa**

**Table 2:** Mean values (± standard deviation) of the nutritional content in leaves of *M. obtusifolia, M. subsericea* and *I. laurina* in restinga and forest ecosystems. Asterisks indicate significant differences between sites (T-test, *p≤0.05*).

Species	Ecosystem	N	P	C	C/N
		g/Kg			
*M. obtusifolia*	Restinga	12.66±1.43	0.55±0.09	202.20±10.62	16.19±2.60
	Forest	15.50±0.85*	0.64±0.06	215.40±8.17	13.92±0.66
*M. subsericea*	Restinga	13.70±0.79	0.56±0.09	222.60±6.89	16.30±1.39*
	Forest	15.67±0.46*	0.61±0.03	221.40±7.93	14.15±0.90
*I. laurina*	Restinga	25.58±0.58*	0.83±0.04	221.40±11.98	8.67±0.66
	Forest	23.98±0.67	0.83±0.08	216.00±4.38	9.02±0.44

**Table 3:** Mean values (± standard deviation) of nutritional and physical-chemical characteristics of the soil in restinga and forest. SB – sum of bases; T – potential cation exchange capacity; t – effective cation exchange capacity; m – aluminum saturation percent; V – bases saturation percent at pH 7.0. Asterisks indicate significant differences between sites (T-test, *p≤0.05*).

Soil Characteristic	Restinga	Forest
P (mg dm^−3^)	4.00 ± 1.41	8.60 ± 4.77
K (mg dm^−3^)	17.20 ± 4.09	159.60 ± 54.56*
Ca (cmol_c_ dm^3^)	0.50 ± 0.22	1.60 ± 0.87*
Mg (cmol_c_ dm^3^)	0.18 ± 0.04	1.74 ± 1.06*
Na (cmol_c_ dm^3^)	0.08 ± 0.03	0.53 ± 0.27*
C (%)	0.85 ± 0.05	2.98 ± 1.34*
N (%)	0.09 ± 0.03	0.20 ± 0.08*
Al (cmol_c_ dm^3^)	0.16 ± 0.09	0.12 ± 0.31
SB (cmol_c_ dm^3^)	0.80 ± 0.22	4.26 ± 2.18*
T (cmol_c_ dm^3^)	2.48 ± 0.31	10.44 ± 3.15*
t (cmol_c_ dm^3^)	0.96 ± 0.13	4.46 ± 1.96*
m (%)	7.64 ± 9.89	7.16 ± 11.92
V (%)	32.54 ± 7.59	38.98 ± 8.66

The raw data presents the unprocessed data used from ecophysiology, and leaf and soil nutritional analyses. The raw data from the gas exchange (*A* µmol m*^−2^* s*^−1^; E mol* m*^−2^* s*^−1^*; and gs mmol m*^−2^* s*^−1^*) and chlorophyll *a* fluorescence (*YII and Fv/Fm*) present values which were performed in two times (8 am and 12 pm) to 25 leaves per species in both ecosystems (Restinga and Forest). The raw data values of the nutritional content in leaves refer to 4 leaves by species in the two ecosystems. For the physical-chemical soil characteristics the data refers to five composite samples collected in both ecosystems. The raw data is shared as supplementary material in the Mendeley Data repository.

## 2 Experimental design, materials and methods

### 2.1 Sampling location and plant material

The samples were obtained in a restinga (21°79’71” S, 41°04’25” W) and in a seasonal semideciduous forest (21°41’57” S, 41°07’76” W), both located in the northern region of Rio de Janeiro - Brazil. Despite the proximity (± 40 km in straight-line), the microclimate and edaphic conditions between the two locations are very contrasting, as presented by Pireda et al. [1] and in Fig. 3.

The species selection, *Maytenus obtusifolia* Mart. (Celastraceae), *Manilkara subsericea* (Mart.) Dubard (Sapotaceae) and *Inga laurina* (Sw.) Willd. (Fabaceae) (Fig. 3 J – O) was based in three criteria: co-occurrence at both sites, high importance value index (IVI); and relative frequency (RF) [2,3]. For each species, five individuals were selected per area, of which five fully expanded leaves were collected from the third node.

The analyses were performed during the rainy season (January and February) of 2017.

### 2.2 Light microscopy

The light microscopy was used to obtain data regarding the leaf anatomical characterization. For this purpose, three leaves were selected by individuals, from which fragments of the leaf middle third were removed. These fragments were fixed in a solution of 2.5% glutaraldehyde, 4% formaldehyde and 0.05M sodium cacodylate buffer at pH 7.2 [4]. Subsequently, the material was post-fixed in 1% osmium tetroxide and 0.05M sodium cacodylate buffer for 2 h and dehydrated in ascending series of acetone. After dehydration, the material was infiltrated and embedded in epoxy resin (Epon®) and taken to a drying oven at 60°C for polymerization and obtaining the blocks. The blocks were placed in ultramicrotome (Reichert Ultracuts Leica Instruments®), from which semi-thin sections (70 nm) were obtained. These sections were stained in 1% toluidine blue and borax buffer [5], and observed under a light microscope (Axioplan, ZEISS, Germany), coupled to an image capture system (Moticam Pro 282B, Hong Kong).

### 2.3 Gas exchange

The gas exchange data were obtained during the day (8 am and 12 pm) with a portable infrared carbon dioxide analyzer (LCpro-SD, ADC BioScientific Ltd., UK). To perform the measurement, 25 leaves per species were selected, divided between five individuals, in both sites (*n* = 50). The irradiance conditions of the camera were adjusted to 2000 µmol m^−2^ s^−1^, and environmental conditions were used for measures of temperature and humidity. The CO_2_ capture was performed with an air probe, positioned 2 m from the soil. The following parameters were analyzed: liquid photosynthetic rate (*A µmol* m*^−2^* s*^−1^*), transpiration rate (*E mol* m*^−2^* s*^−1^*) and stomatal conductance (gs mmol m*^−2^* s*^−1^*).

### 2.4 Chlorophyll a fluorescence

The chlorophyll *a* fluorescence was performed to obtain data related to photosystems II performance. For this, the same leaves selected for gas exchange were used. These leaves were initially adapted to dark with the aid of leafclips for approximately 30 min. The leaves were first exposed to low intensity modulated red light (6 µmol m^−2^s^−1^ at 660 nm), and then exposed to high intensity actinic white light (10,000 µmol m^−2^s^−1^) applied for 0.8 s [6]. The data were obtained with a portable fluorimeter (OS5p Opti-Sciences, UK). The observed variables were maximum quantum yield of PSII (*F_v_/F_m_*) and quantum efficiency of PSII (*YII*).

### 2.5 Leaf nutritional analysis

The nutritional content in leaves was obtained from 500 g of leaves per individual, a total of n = 10 per species. The leaves were initially dried in a drying oven at 70°C and after were taken to quantify the C, N, and P content in the analysis center of Universidade Federal Rural do Rio de Janeiro, Campus – Campos dos Goytacazes, Brazil. The data were obtained using the methodology of Embrapa 2000 [7].

### 2.6 Nutritional and physical analysis of the soil

For the nutritional and physic-chemical analysis of the soil, four soil samples were collected using a probe and close to five individuals of each species. These samples were subsequently homogenized, obtaining a total of 15 composite samples. The analyses were performed at Universidade Federal Rural do Rio de Janeiro, Campus – Campos dos Goytacazes, Brazil, following the methodology of Embrapa 1997 [8].

### 2.7 Statistical analysis

The normality of the data was assessed using the Shapiro-Wilk test when *n* < 30 and the Kolmogorov-Smirnov test when n > 30. Levene's test was used to assess the homogeneity of the data. T-test (*p* < 0.05) was used for the estimation of the significances between means of nutritional content in leaves and the soil physical-chemical characteristics, and ANOVA followed by the Tukey test (*p* < 0.05) were used to assess gas exchange and chlorophyll *a* fluorescence. The statistical analysis was performed using Statistica7 software.

## Declaration of Competing Interest

The authors declare that they have no known competing financial interests or personal relationships which have, or could be perceived to have, influenced the work reported in this article.

## Appendix Supplementary materials

Image, application 1
